# Singular Species of Epizoa

**Published:** 1867-12

**Authors:** N. B. Drewry

**Affiliations:** Griffin, Ga.


### ARTICLE III.

*Singular Species of Epizoa.* By N. B. Drewry, M. D., Griffin, Ga.

In July of the present year, Mrs. B——, æt. 28 years, of full habit and florid complexion, and active life, applied to J. R. Cleaveland, S. D., of this place, for relief of tooth-ache. Finding a cavity, he proceeded to treat the nerve by the application of arsenic. Failing to allay the pain in this way, he resorted to mechanical means of impressing or destroying the nerve, and was finally successful in relieving the suffering. Very soon, however, the pain returned, and as it was desirable to avoid extraction, if comfort could be obtained otherwise, the instrument was again introduced into the cavity of the tooth, and, after giving it a rotary motion, was removed. On examination of the substance adhering to the instrument, a living animal—a worm—was found. The parisite measures about four lines in length and one in thickness, with a head larger than the transverse measurement of the body, and composed of a firm, horny substance. Since the removal of the worm from its cavity, the tooth has not been at all painful.

A still more remarkable case of local disturbance by an epizoon, presented itself to me, one month after the case above-mentioned was treated.

Mrs. G——, æt. about 43, healthy and corpulent, called,

August 2, 1867, at my office to consult me in regard to a painful affection of the finger, very much resembling ordinary whitlow. The pain, she said, was periodical, deep-seated and of a gnawing character. Seeing nothing unusual in these symptoms, I pronounced the case a felon, and gave the usual advice in such cases. She declined, for the time, having it opened, but applied emolients, used opiates internally, etc. Two weeks passed in this way, without any material change in the condition of the part, except a slight pointing at the center of swelling. Pricking this portion of the cuticle, with an ordinary needle, she imagined, though without much discharge, gave some comfort. With the hope of obtaining entire relief in this way, she still refused to have the finger laid open with a bistoury. In two or three days after her own operation with the needle, the suffering became again so intense, that she resorted to the same mode of relief. Extending her puncture, perhaps, further than before, she found beneath the attenuated skin a living worm, and extracted it. The pain subsided at once, and the swelling, and other evidences of local disease, gave way.

The worm thus extracted, and to which the painful condition of the finger is attributable, in every respect, resembles that obtained from the tooth of Mrs. B——, a month previously.

